# Optimal antithrombotic treatment of patients with atrial fibrillation undergoing percutaneous coronary intervention: triple therapy is too much!

**DOI:** 10.1007/s12471-018-1120-6

**Published:** 2018-05-08

**Authors:** M. S. Jacobs, R. G. Tieleman

**Affiliations:** 1 Department of Clinical Pharmacy and Toxicology, Martini Hospital, Groningen, The Netherlands; 2 Groningen Research Institute of Pharmacy, Unit of PharmacoTherapy, Epidemiology & Economics (PTEE), University of Groningen, Groningen, The Netherlands; 3 Department of Cardiology, Martini Hospital, Groningen, The Netherlands; 4 Department of Cardiology, University Medical Center Groningen, University of Groningen, Groningen, The Netherlands

**Keywords:** Anticoagulation, Antiplatelet therapy, Atrial fibrillation, Acute coronary syndrome, Coronary intervention

## Abstract

Patients with atrial fibrillation who undergo a coronary intervention are eligible for both anticoagulation and (dual) antiplatelet therapy ((D)APT). An optimal balance has to be found to reduce the thromboembolic risk (i.e. stroke, systemic embolism and myocardial infarction) and to minimise the increased risk of bleeding with concomitant use of an anticoagulant and (D)APT. Owing to a lack of evidence, the guideline recommendations are predominantly based on expert opinion. Current evidence indicates that the combination of a non-vitamin K oral anticoagulant (NOAC) and clopidogrel is safer than vitamin-K oral antagonists plus DAPT, which increases the risk of bleeding, without clear advantages in regard to efficacy. Concerning whether (*N*)OACs should be combined with single APT rather than DAPT, the findings of the WOEST, PIONEER AF-PCI and RE-DUAL PCI trials seem to favour a combination with clopidogrel only, thus omitting aspirin. Choosing the optimal treatment strategies for individual patients on NOACs and (D)APT will remain a challenge for clinicians, though triple therapy seems to be the less favourable option owing to the increased risk of bleeding.

## Introduction

Almost 30% of patients with atrial fibrillation (AF) have co-existing coronary artery disease (CAD) [1, 2]. Anticoagulants are prescribed to AF patients to reduce the risk of stroke or systemic embolism. However, there is still a risk of the patient developing acute coronary syndrome (ACS), requiring percutaneous coronary intervention (PCI). Following PCI, antiplatelet drugs such as aspirin and P2Y12 inhibitors (clopidogrel, prasugrel and ticagrelor) play a predominant role in the prevention of in-stent thrombosis and cardiovascular events [3]. Antiplatelet therapy (APT) alone is inferior to oral anticoagulation for reducing the risk of thromboembolic events in AF patients [4]. According to the present European Society of Cardiology (ESC) guidelines, AF patients with co-existing ACS and those who undergo a coronary intervention are eligible for both anticoagulation and (dual) antiplatelet therapy ((D)APT) [5, 6]. However, a combination of warfarin with (D)APT carries a more than threefold higher risk for non-fatal and fatal bleeding compared with warfarin monotherapy [7]. An optimal balance has to be found to reduce the thromboembolic risk (i.e. stroke, systemic embolism and myocardial infarction) and to minimise the increased risk of bleeding resulting from concomitant use of an anticoagulant and (D)APT. Owing to the lack of evidence, the 2016 ESC guideline on the treatment of ACS in AF patients and the 2017 ESC focused update on DAPT in CAD are predominantly based on expert opinion [3, 6, 8]. This review summarises and discusses the most recent key developments and future studies with regard to combining anticoagulation (non-vitamin K oral anticoagulants (NOACs) or vitamin K antagonists (VKA)) with APT in AF patients undergoing PCI.

**Figure:**
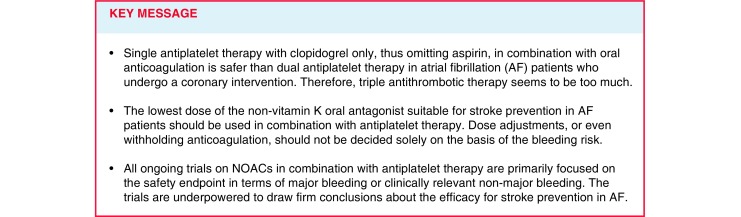

## Guideline recommendations and current practice

The 2014 AF guideline for the management of AF patients of the American College of Cardiology/American Heart Association Task Force on Practice Guidelines and the Heart Rhythm Society (AHA/ACC/HRS) recommends warfarin rather than NOACs as the first-choice therapy in AF patients with ACS. This guideline mentions considering dual therapy consisting of an OAC plus clopidogrel 75 mg once daily (q.d.) as an alternative to initial triple therapy [5].

The 2015 ESC non-ST segment elevation myocardial infarction (NSTEMI) guideline recommends that the use of prasugrel or ticagrelor as a part of triple therapy should be avoided in the absence of safety and efficacy data (class C recommendation) [3]. Because it is known that the addition of (D)APT to oral coagulation increases the bleeding risk, limited data suggest that clopidogrel is probably the safest of the available P2Y12 inhibitors because of its lowest bleeding risk [9]. If the OAC is a VKA, the ESC NSTEMI guideline recommends a target international normalisation ratio (INR) of 2.0–2.5 with the exception of patients with a mechanical prosthetic valve in the mitral position [3].

The 2016 ESC guideline for AF recommends a short period of triple therapy (OAC + aspirin + clopidogrel) followed by a period of dual therapy (OAC + APT, preferably up to 12 months after the event) in AF patients with co-existing ACS and those who undergo PCI [6]. The duration of the dual and triple therapy depends on the bleeding risk as calculated by the HAS-BLED score, the reason for the PCI (ACS or stable CAD) and the type of stent (bare-metal versus drug-eluting). Modifiable bleeding risk factors should be corrected to minimise the risk of bleeding. This guideline also mentions dual therapy with an OAC and clopidogrel as an emerging alternative to triple therapy based on the results of the WOEST study (What is the Optimal antiplatElet and anticoagulant therapy in patients with oral anticoagulation and coronary StenTing) [10] When a NOAC is preferred for anticoagulation, the lowest effective dose for stroke prevention should be used (apixaban 5 mg twice daily (b.i.d.), dabigatran 110 mg b.i.d., edoxaban 60 mg q.d., rivaroxaban 20 mg q.d.) or the appropriate reduced dose if indicated by dose-reduction criteria according to the drug labelling [2].

Fairly recently, a new ESC focused update was published on DAPT in CAD [8]. The guideline expresses a preference for clopidogrel as a part of triple antithrombotic therapy; prasugrel and ticagrelor should be avoided in combination with oral anticoagulation because of worrisome bleeding rates in registries (level C recommendation). In patients treated with a NOAC, the lowest effective dose for stroke prevention is also recommended in this guideline. Interestingly, the most innovative recommendation in this guideline is the use of rivaroxaban 15 mg q.d. as an alternative to rivaroxaban 20 mg q.d. when combined with aspirin and/or clopidogrel. The new guideline seems to base this level B recommendation on the results of the PIONEER AF-PCI (Open-Label, Randomized, Controlled, Multicenter Study Exploring Two Treatment Strategies of Rivaroxaban and a Dose-Adjusted Oral Vitamin K Antagonist Treatment Strategy in Subjects with Atrial Fibrillation who Undergo Percutaneous Coronary Intervention) study. It is noteworthy that this same guideline states that the PIONEER AF-PCI study is largely underpowered for the assessment of meaningful differences in the incidence of relevant ischaemic events, such as stent thrombosis or stroke rates. Dual therapy with clopidogrel 75 mg and oral anticoagulation is still recommended for consideration as an alternative to 1 month of triple therapy for patients in whom the bleeding risk outweighs the ischaemic risk [8].

The European Heart Rhythm Association conducted a survey in 2014 to provide an insight into current practice in Europe regarding the management of AF patients presenting with ACS. This survey showed that, in 91.1% of cases, combining warfarin with aspirin and clopidogrel was preferable in AF patients after PCI [11]. Most of the responding centres treated patients with triple therapy (OAC + aspirin + clopidogrel) for 3 months followed by 12 months of dual therapy (OAC + aspirin), and the majority used warfarin [11, 12]. Of the NOACs, rivaroxaban 15 mg o.d. and apixaban 2.5 mg b.i.d. were most frequently used.

## Triple vs dual antithrombotic therapy

### Post hoc analyses

In the post hoc analysis of the RE-LY (Randomized Evaluation of Long-Term Anticoagulation Therapy) trial, dabigatran 110 mg b.i.d. plus (D)APT was not inferior to warfarin plus (D)APT in reducing stroke and systemic embolism and was associated with fewer major bleeds. There was an additive effect on major bleeding risk with the number of antiplatelet drugs used regardless of the OAC dose used, but (D)APT did not reduce the risk of thromboembolism or stroke [13]. A subanalysis of the ENGAGE AF-TIMI 48 (Effective Anticoagulation with Factor Xa Next Generation in Atrial Fibrillation—Thrombolysis in Myocardial Infarction 48) trial also looked at the combination of a NOAC plus APT [14]. Concomitant use of edoxaban 60 mg q.d. or 30 mg q.d. plus APT was compared with warfarin plus APT in AF patients. The study showed that combining APT with edoxaban or warfarin increased the bleeding risk. There was less major bleeding in patients that used edoxaban than in those using warfarin. Moreover, APT did not influence the efficacy of edoxaban compared to warfarin in preventing stroke or systemic embolic events. A predefined analysis of the ARISTOTLE (Apixaban for Reduction in Stroke and Other Thromboembolic Events in Atrial Fibrillation) trial assessed the effect of concomitant aspirin use on the efficacy and safety of apixaban compared with warfarin in AF patients [15]. Apixaban use resulted in similar reductions in stroke and systemic embolism among aspirin users and non-users, compared with warfarin. Apixaban had a consistent effect among aspirin users and non-users on stroke or systemic embolism, ischaemic stroke, myocardial infarction, and death both in the overall population and among the subgroups of patients with and without a history of arterial vascular disease. There were statistically significant reductions in major bleeding for aspirin combined with apixaban compared to warfarin plus aspirin. The effect of apixaban in causing less major bleeding, haemorrhagic stroke, major or clinically relevant non-major bleeding and any bleeding was comparable in subgroups with and without a history of arterial vascular disease. Only 0.7% of the total population of the ARISTOTLE trial underwent triple therapy (aspirin + P2Y12 receptor antagonist + study drug) for at least 7 days. There was no subanalysis for AF patients with ACS or patients who underwent PCI.

### Results of (N)OACs combined with (D) APT in AF patients

The WOEST study compared the safety of triple therapy (VKA (target INR 2.0) with clopidogrel and aspirin) with that of dual therapy (VKA with clopidogrel) in patients that used an OAC and underwent PCI. This study showed that omitting aspirin significantly reduced bleeding complications (hazard ratio (HR) 0.36, 95% confidence interval (CI) 0.26–0.50, *p* < 0.0001) and was associated with a lower risk of the combined secondary endpoint consisting of death, myocardial infarction, stroke, target-vessel revascularisation and stent thrombosis (HR 0.60, 95% CI 0.38–0.94) [10]. It should be noted that the WOEST study was not powered to detect differences in the occurrence of thrombotic events when aspirin was omitted.

The PIONEER AF-PCI trial [16] compared two different rivaroxaban treatment strategies with a VKA treatment strategy in patients with AF undergoing PCI. The drug regimens were based on the results of the ATLAS ACS 2‑TIMI 51 study (Anti-Xa Therapy to Lower Cardiovascular Events in Addition to Standard Therapy in Subjects with Acute Coronary Syndrome-Thrombolysis in Myocardial Infarction 51) and the results of the WOEST study. The PIONEER AF-PCI included the more potent P2Y12 inhibitors ticagrelor and prasugrel as a treatment option, with the choice of the P2Y12 inhibitor at the discretion of the physician. However, over 93% of the patients were treated with clopidogrel, and therefore no statements per type of P2Y12 inhibitor can be made. The PIONEER trial results demonstrated that use of rivaroxaban 15 mg q.d. plus a P2Y12 inhibitor for 12 months and rivaroxaban 2.5 mg b.i.d. plus DAPT for 1, 6 or 12 months was associated with lower rates of clinically significant bleeding compared to VKA plus DAPT for 1, 6 or 12 months (16.8% for rivaroxaban 15 mg q.d. plus P2Y12 inhibitor, 18.0% for rivaroxaban 2.5 mg b.i.d. plus DAPT, 26.7% for VKA plus DAPT; HR = 0.59, 95% CI = 0.47–0.76 for rivaroxaban 15 mg q.d. plus P2Y12 inhibitor vs VKA + DAPT and HR = 0.63, 95% CI = 0.50–0.80 for rivaroxaban 2.5 mg b.i.d. plus DAPT vs VKA + DAPT). The rates of the pre-specified endpoint death from cardiovascular causes, myocardial infarction or stroke were similar in the three groups [16]. Notably, although the stroke rate was not different in the three groups, the study was underpowered to draw any conclusion about the effect on stroke prevention, and the 2.5 mg dose of rivaroxaban was not tested for stroke prevention in AF. The post hoc analysis of the PIONEER AF-PCI trial demonstrated that AF patients who underwent PCI and were treated with rivaroxaban 15 mg q.d. plus a P2Y12 inhibitor for 12 months or rivaroxaban 2.5 mg b.i.d. plus DAPT for 1, 6 or 12 months had a lower risk of all-cause mortality or recurrent hospitalisation for adverse events compared to those with VKA plus DAPT for 1, 6 or 12 months [17].

The RE-DUAL PCI trial (Randomized Evaluation of Dual Therapy with Dabigatran vs Triple Therapy with Warfarin in Patients with Atrial Fibrillation That Undergo a Percutaneous Coronary Intervention with Stenting) compared dual therapy consisting of dabigatran (110 mg and 150 mg b.i.d.) and clopidogrel or ticagrelor with triple therapy consisting of warfarin, aspirin and clopidogrel or ticagrelor in patients with non-valvular atrial fibrillation that underwent a PCI with stenting [18]. In this trial, no distinction is made between the specific P2Y12 inhibitors and the duration of (D)APT treatment, and only 12% received ticagrelor. The average age of the patients was 70.8 years, and ACS was the most frequent PCI indication (50.5%). The incidence of major or clinically relevant non-major bleeding events during follow-up was significantly lower for dabigatran plus P2Y12 than for triple therapy (HR 0.52, 95% CI 0.42–0.63 for dabigatran 110 mg and HR 0.72, 95% CI 0.58–0.88 for dabigatran 150 mg). The two dual-therapy groups were not inferior with respect to the incidence of the composite efficacy endpoint (myocardial infarction, stroke, systemic embolism, death or unplanned revascularisation) as compared with the triple-therapy group. In contrast to the PIONEER AF-PCI trial, the NOAC doses in the RE-DUAL PCI trial (dabigatran 150 and 110 mg b.i.d.) have both been proved to be effective for stroke prevention in AF. Unfortunately, as only a small number of patients were enrolled per dabigatran dose group, the trial was not powered to examine the efficacy according to dose. Also, the trial was not powered to allow for comparisons of individual components of the efficacy endpoint.

Overall, the results from the WOEST, PIONEER AF-PCI and the RE-DUAL PCI trials suggest that in AF patients who recently underwent PCI, dual therapy with a combination of (N)OAC and clopidogrel is safer than triple therapy with OAC plus aspirin and clopidogrel. The post hoc analysis of the RE-LY study demonstrated that dabigatran 110 mg b.i.d. plus DAPT (aspirin and clopidogrel) is safer than VKA plus DAPT in terms of bleeding. Whether this is also true for dabigatran plus clopidogrel versus VKA plus clopidogrel cannot be concluded from the study results published thus far. It should be noted that the RE-DUAL and PIONEER AF-PCI studies as well as the post-analyses of previous studies had a target INR of 2.0–3.0, which is higher than the guideline recommendation of 2.0–2.5. The time in therapeutic range (TTR) is a very import aspect to take into account, since a low TTR could influence the clinical outcomes.

### Ongoing trials

A summary of the multiple ongoing trials evaluating the role of NOACs and (D)APT in AF patients with co-existing ACS is presented in Tab. 1. The AUGUSTUS trial (an Open-label, 2 × 2 Factorial, Randomized Controlled, Clinical Trial to Evaluate the Safety of Apixaban vs Vitamin K Antagonist and Aspirin vs Aspirin Placebo in Patients with Atrial Fibrillation and Acute Coronary Syndrome or Percutaneous Coronary Intervention; NTC02415400) compares the safety of apixaban 5 mg (or 2.5 mg) b.i.d. with VKA both with and without aspirin in patients with AF and ACS or PCI. All patients in this study will also be taking a P2Y12 inhibitor. Because of its 2 × 2 factorial design, this study will provide insight into the safety of apixaban versus a VKA when both are combined with single APT. Moreover, it will address the safety and efficacy of triple versus dual therapy in a randomised design: OAC (VKA/NOAC) with DAPT versus OAC (VKA/NOAC) with single APT. The safety and efficacy of edoxaban in AF patients who undergo PCI with stenting will be investigated in the ENTRUST-AF-PCI trial (Edoxaban Treatment Versus Vitamin K Antagonist in Patients with Atrial Fibrillation Undergoing Percutaneous Coronary Intervention; NTC02866175). This study will compare the safety and efficacy of 60 mg or 30 mg edoxaban plus clopidogrel or another P2Y12 inhibitor with a VKA plus clopidogrel and 1–12 months of aspirin (in the presence of a documented clinical need: prasugrel or ticagrelor) [19]. The ongoing trial investigating rivaroxaban in combination with APT in AF patients with ACS is the RT-AF trial (Rivaroxaban in Patients with Atrial Fibrillation and Coronary Artery Disease Undergoing Percutaneous Coronary Intervention; NTC02334254). The RT-AF trial evaluates the safety of rivaroxaban 2.5 mg b.i.d. plus ticagrelor versus triple therapy with warfarin plus clopidogrel and aspirin [20]. Safety is assessed based on a composite primary endpoint of major bleeding and clinically relevant non-major bleeding. The last ongoing trial which will provide information about the management of NOACs in AF patients with ACS is the WOEST 2 registry (NTC0263520). This study is a prospective, international registry on concomitant use of OACs and P2Y12 inhibitors in patients with AF or heart valve prosthesis undergoing coronary revascularisation. This study will provide insight into the efficacy and safety of all possible combinations of NOACs and antiplatelet inhibitors.

**Table 1:** Overview of ongoing trials on concomitant use of non-vitamin K oral anticoagulants and antiplatelet drugs in atrial fibrillation patients with an acute coronary syndrome

Trial	AUGUSTUS	ENTRUST-AF-PCI	RT-AF	WOEST 2 registry
Study design	Open-label, 2 × 2 factorial, randomized controlled clinical trial	Randomized, open label, safety study	Open-label, randomized controlled clinical trial	Prospective, international, multi-center, non-interventional, cohort study
Objective	To assess the safety of the different treatment arms in AF patients undergoing PCI	To assess the safety of the different treatment arms in AF patients undergoing PCI	To assess the safety of the different treatment arms in AF patients undergoing PCI	To assess the different management patterns and safety and efficacy outcomes of combined use of OAC and a P2Y12 inhibitor in patients with AF and/or a heart valve prosthesis undergoing PCI
Pt number	4,600	1,500	420	2,200
Follow-up	6 months	24 months	12 months	24 months
Estimated completion	December 2018	February 2019	January 2016	December 2019
Arms	Apixaban 5 mg (or 2.5 mg according to dose reduction criteria) b.i.d. + P2Y12 inhibitor + ASA 81 mg q.d.	Edoxaban 60 mg q.d. (or edoxaban 30 mg q.d according to dose reduction criteria) + clopidogrel 75 mg q.d.	Rivaroxaban 2.5 mg/5 mg b.i.d. + ticagrelor 90 mg b.i.d. Antiplatelet therapy is mandatory at least 1 month after BMS implantation, and 6 months after DES implantation	All combinations of chronic OAC and a P2Y12 inhibitor with or without ASA
	Apixaban 5 mg (or 2.5 mg) b.i.d. + P2Y12 inhibitor + Placebo	VKA + clopidogrel 75 mg q.d. + ASA 100 mg 30 days to 12 months		
	VKA + P2Y12 inhibitor + ASA 81 mg q.d.	In case of a documented clinical need: prasugrel [5 or 10 mg q.d.] or ticagrelor [90 mg b.i.d.]	Rivaroxaban 2.5 mg/5 mg b.i.d. + ticagrelor 90 mg b.i.d. Antiplatelet therapy is mandatory at least 1 month after BMS implantation, and 6 months after DES implantation	
	VKA + P2Y12 inhibitor + placebo			
Primary outcome	Time to ISTH major or CRNM bleeding during the treatment period	Number of major or CRNM ISTH-bleeding	Number of major or CRNM bleeding	Composite of thrombotic events (MI, stroke, TIA, SE) and cardiovascular death and Bleeding events

*AF* atrial fibrillation, *ASA* aspirin, *b.i.d.* twice daily, *BMS* bare metal stent, *CRNM* Clinically Relevant Non-Major, *DES* drug-eluting stent, *INR* international normalized ratio, *ISTH* International Society on Thrombosis and Hemostasis, *MI* myocardial infarction, *NVAF, OAC* oral anticoagulant, *q.d.* once daily, *PCI* percutaneous coronary intervention, *SE* systemic embolism; *TIA* transient ischemic attack, *VKA* vitamin K antagonist

Most of the treatment arms of the ongoing trials focus on one single NOAC combined with a single antiplatelet drug. Based on the design of the trials, superiority or even non-inferiority of combining a single antiplatelet drug with a NOAC compared to DAPT plus a NOAC cannot be demonstrated, with the exception of the AUGUSTUS trial, where apixaban plus clopidogrel plus aspirin will be compared with apixaban plus clopidogrel plus placebo. Of the ongoing trials, RT-AF is the only one that uses a lower target INR (1.8–2.5). None of these trials are performing a head-to-head comparison of different NOACs and therefore provide no insight into the difference in efficacy and safety of these agents.

## Discussion

All of the ongoing trials are primarily focused on the safety endpoint in terms of major bleeding or clinically relevant non-major bleeding. All of these trials are underpowered to draw firm conclusions about the efficacy for stroke prevention in AF. This is of importance, since some of the trials (i.e. PIONEER AF-PCI and RT-AF) use a suboptimal NOAC dose for stroke prevention in AF. The short follow-up period of all the studies limits their ability to assess the effect on stroke and venous thromboembolic events, especially in the long term. Furthermore, these studies are also underpowered for the clinical endpoints death, myocardial infarction and in-stent thrombosis in patients with ACS. Most of the studies do not include patients who have had a stroke or bleeding, although these events do not contraindicate NOAC use. When determining the optimal treatment for an individual patient, the clinician should always take into account bleeding risk factors. Modifiable bleeding risk factors are, for example: adding a proton pump inhibitor with DAPT to reduce the risk of gastrointestinal bleeding, hypertension (<160 mm Hg target), alcohol use and the use of predisposing co-medication such as non-steroidal anti-inflammatory drugs.

Despite the efforts of researchers to synthesise more evidence from the ongoing trials, there will still be challenges for clinicians to choose the optimal treatment strategies for individual patients on NOACs and (D)APT with AF undergoing PCI. The ESC guidelines and the AHA/ACC/HRS recommendation to use the lowest effective NOAC dose in combination with (D)APT in patients with AF and co-existing ACS or PCI were based on limited evidence concerning the optimal combination of NOACs and APT (level of evidence C; expert opinion) [3, 5, 8]. In this respect, the term “lowest effective dose” could be confusing and the term “lowest suitable dose for AF” would be more appropriate. Current evidence indicates that the combination of NOAC and clopidogrel is safer than VKA plus DAPT, which increases the risk of bleeding, without clear advantages in efficacy.

As to whether (N)OAC should be combined with single APT rather than dual APT, evidence from the WOEST, PIONEER AF-PCI and RE-DUAL PCI trials seems to favour a combination with clopidogrel only, thus omitting aspirin [21]. In this respect, a very interesting trial would involve a treatment arm with only an OAC in an adequate dose for stroke prevention in AF without APT. Unfortunately, this has not been incorporated into any of the studies. A direct comparison of clopidogrel versus aspirin as APT after PCI has not been studied. Evidence from a randomised controlled trial in patients with atherosclerotic vascular disease (manifested as either ischaemic stroke, myocardial infarction or symptomatic peripheral artery disease) showed that clopidogrel was more effective than aspirin in reducing the combined risk of ischaemic stroke/myocardial infarction and vascular death [22]. A nationwide registry study confirmed that there was a marginally significant reduction of MI and coronary death with OAC plus clopidogrel compared to triple therapy in AF patients after myocardial infarction or PCI. This reduction was not significant for OAC plus aspirin [23]. Also, OAC plus aspirin was associated with a significantly increased risk of death from all causes. The choice of clopidogrel over aspirin in ongoing trials seems to be based mainly on the WOEST trial results. None of the studies thus far has made a direct comparison of a VKA plus single APT versus a NOAC plus single APT. The upcoming results of the AUGUSTUS trial will therefore be interesting owing to its 2 × 2 factorial design (see Tab. 1). The reduced-dose NOACs should only be given if the dose reduction is indicated according to the patients’ characteristics as listed in the drug labelling, irrespective of the APT used. A reduced-dose NOAC in “healthy” patients, with the exception of dabigatran 110 mg q.d., has not been shown to be effective for stroke prevention in AF. A common mistake is to give apixaban 2.5 mg combined with APT instead of the full dose. However, this would only be appropriate in the presence of dose-reduction criteria (creatinine clearance 10–30 ml/min or two out of three following: serum creatinine ≥133 µmol/l, body weight ≤60 kg and/or age ≥80 years). The recommendation of the 2017 ESC guideline on DAPT to consider rivaroxaban 15 mg q.d. instead of 20 mg when combined with aspirin and/or clopidogrel should therefore be followed with caution because this dose has not been proved to be effective for stroke prevention in patients with AF. Consequently, rivaroxaban 2.5 mg is not an option for stroke prevention in ACS patients with AF.

The majority of the above-mentioned NOAC trials are still recruiting at present, and the results will become available in the coming years. For now, triple therapy has proved to be too much in terms of safety outcomes. However, it can be expected that guidelines will continue to change in the near future, and what seems wise today may become obsolete tomorrow.
